# 

**Published:** 1894-04-28

**Authors:** 


**Notices of Births, Deaths, and Marriages** are inserted in this column at a charge of 2s. 6d. for an announcement not exceeding four lines.

MARRIAGE.

On April 19th, at St. Alphage Church, Greenwich, by the Rev. Brooke Lambert, M.A., B.C.L., assisted by the Rev. C. E. Escrut, M.A., Rector of St. Mary, Woolwich, Courtenay James Fuller, M.R.C.S., L.R.C.P., of 37, Rectory Place, Woolwich, to Clara Cornwall, youngest daughter of Prior Purvis, M.D. (Lond.), of 5, Lansdowne Place, Blackheath. At home (Rectory Place) on each Monday after June 1st.

**Notice to Advertisers and Subscribers.**

All Advertisements, Orders for Copies of The Hospital, and Business Communications should be addressed to **The Publisher, NOT TO THE EDITOR,** The Hospital, 428, Strand, W.C.

The Cost of Subscription, post free, is as follows:—Per week, 2½d.; per quarter, 2s. 8d.; per half-year, 5s. 3d.; per annum, 10s. 6d. Foreign:—Per Annum, 12s. 8d.; payable, in all cases, in advance.

### NOTICE TO CORRESPONDENTS.

All MS., Letters, Books for Review, and other matters intended for the Editor should be addressed The Editor, The Lodge, Porchester Square, London, W.

The Editor cannot undertake to return rejected MS., even when accompanied by stamped directed envelope.

**"Burdett's Hospital Annual," 1894.**

Fifth year of publication. 900 pp. Boards, gilt lettering. A most complete guide to British, Colonial, Indian, and American Hospitals, Dispensaries, Nursing and Convalescent Institutions, and Asylums. Price 5s. Published by The Scientific Press (Limited), 428, Strand, W.C.

**NOTICE.—The Index for Vol. XIV. (ending Sept. 30th, 1893) is on sale at the Office of this Journal, price 2½d., post free.**

### Books Received.

John Ball and Sons.

"Congenital Affections of the Heart." By George Carpenter, M.D. Lond.

Rivington, Percival, and Co.
"Health at School." By Clement Dukes, M.D.

Cassell and Co.
"Difficult Labour." By G. Ernest Herman.

W. T. Keener Company, Chicago.
"Electricity in Diseases of Women and Obstetrics." By Franklin H. Martin, M.D.

Bailliere, Tindall, and Co.

"Nature's Hygiene." By C. T. Kingzelt, F.S.C.

Scientific Press.
"Mental Nursing." By William Harding, M.D.
"Hospitals, Dispensaries, and Nursing." Edited by John S. Billings, M.D., and Henry M. Hind, M.D.

Sampson Low and Co.
"A Manual of Obstetric Nursing." By Marian Humfrey.

### Scraps and Gleanings.

The Clothworkers' Company have sent a cheque of £50 to the Chelsea Hospital for Women.

The financial statement of the Jeovil Cottage Hospital presented at the last annual meeting shows an adverse balance of £62.

The Worshipful Company of Clothworkers has sent a donation of £60 to the Royal Hospital for Diseases of the Chest, City Road, E.C.

The financial statement of the Belfast Ophthalmic Institution is a favourable one, the income having augmented during the past year.

The first annual meeting of subscribers to the Cotswold Convalescent Home bears testimony to an excellent beginning of the work the institution has undertaken.

The work of the Glasgow Cancer Hospital has been carried on most successfully during the year, 203 patients being received for treatment. A good credit balance now exists on the hospital funds.

A meeting of the General Committee of the Birmingham New General Hospital has just been held, when the tender of Messrs. Barnsley, a local firm, was accepted for the erection of the building at a cost of £117,888.

The Marquis of Zetland has issued a letter in which he states that he has consented to take the chair at the festival dinner of the East London Hospital for Children, which will be held at the Hotel Metropole on May 7th.

At the annual meeting of the directors of the Edinburgh Royal Maternity and Simpson Memorial Hospital, the abstract of accounts showed an income of £888 and an expenditure of £701, leaving a balance in hand of £187.

At the yearly general meeting of the Caisse des Pensions de Retraite du Corps Médical Francais the report, read by the treasurer, stated that up to March 31st, 1894, £19,000 had been paid in. This year the retiring pensions will be paid.

Dr. Sinclair Coghill, senior physician to the Royal National Hospital, Ventnor, has received from his Majesty King Alexander the diploma and insignia of the fourth class Knight Commander of the Royal Servian order of St. Salve.

In accordance with a suggestion made at the last annual meeting, a subscription was set on foot for providing a musical instrument for the Surbiton Cottage Hospital. The appeal was most successful, and a piano has now been placed in the men's ward.

It was pointed out that there existed a grave necessity for having this institution endowed with such a capital fund as would give greater financial stability than was possible with the precarious subsistence to be obtained from annual subscriptions alone.

A meeting, representing over 3,000 members of the various friendly societies of Hanley, met recently together to pass a hearty resolution of thanks to the Messrs. Hanley in recognition of their munificent gift of £20,000 for the erection of a convalescent home for North Staffordshire.

A bazaar has lately been organised at Durham, promoted by those interested in the Private Fever Hospital at Houghall. One hundred pounds was required to meet the wants of the hospital, towards which the net proceeds of the bazaar, after paying all expenses, contributed £72.

In proof that the Bristol Children's Hospital is appreciated by parents whose children need medical or surgical treatment, it is only necessary to state that during the past year 860 little ones have been under care as in-patients of the hospital, whilst 2,954 have been prescribed for as out-patients.

The National Lying-in Hospital, Holles Street, Dublin, has been formally re opened. At this ceremony a letter from the Archbishop was read, promising the munificent contribution of £1,000, in the form of a subscription of £100 a year for the first ten years of the working of the institution.

### Medical Vacancies and Appointments.

### VACANCIES.

### Resident Medical Officers.

Dewsbury and District General Infirmary.—House Surgeon, doubly qualified. Salary commencing at £80 per annum, with board and residence. Applications, endorsed "House Surgeon," to the Chairman of the House Committee, Infirmary, Dewsbury, by May 1st.

East London Hospital for Children, Shadwell, E.—House Surgeon. Board and residence provided, no salary. Applications to Thomas Hayes, Secretary, by May 5th.

Flintshire Dispensary.—House Surgeon. Salary £120 per annum, with furnished house, rent and taxes free; also coal, light, water, and cleaning, or, in lieu thereof, £20 per annum. Knowledge of Welsh desirable. Applications to W. T. Cole, Secretary, Board Room, Bagillt Street, Holywell, by May 15th.

General Hospital, Birmingham.—Assistant House Surgeon. Appointment for six months. Residence, board, and washing provided. No salary. Applications to the House Governor by April 28th.

North Staffordshire Infirmary and Eye Hospital, Hartshill, Stoke-upon-Trent.—Assistant House Surgeon. Board, apartments, and washing provided. Applications to the Secretary by May 1st.

Royal Victoria Hospital, Bournemouth.—House Surgeon and Secretary. Salary £100 per annum, with board. Applications to the Chairman of Committee by May 1st.

### Non-Resident Medical Officers.

Balrothery Union, Swords Dispensary.—Medical Officer. Salary £125 per annum, with £20 16s. 8d. as Medical Officer of Health, together with registration and vaccination fees. Applications to Mr. Michael Long, Honorary Secretary. Election on May 1st.

Dental Hospital of London and London School of Dental Surgery, Leicester Square.—Demonstrator. Honorarium £50 per annum. Applications to Morton Smale, Dean, by May 14th.

Dental Hospital of London, Leicester Square. — Two Assistant Anæsthetists. Applications to J. Francis Pink, Secretary, by May 14th.

London Hospital, Whitechapel, E.—Medical Registrar. Salary £100 per annum. Applications to the House Governor by May 5th.

St. Marylebone General Dispensary, 77, Welbeck Street, Cavendish Square, W.—Honorary Physician. Applications to the Secretary by April 30th.

Salford Royal Hospital.—Honorary Medical Officer for the Pendleton Branch Dispensary, doubly qualified. Applications to the Secretary by April 28th.

Westminster Hospital, Broad Sanctuary, S.W.—Surgical Registrar. Must be F. or M.R.C.S.Eng. Appointment for twelve months. Salary £40 per annum. Applications to Sidney M. Quennell, Secretary, by May 22nd.

### The Hospital Advertiser.

### BOOKS FOR NURSES.

Demy 8vo., 200 pp., cloth extra, price 3s. 6d.

**SURGICAL WARD-WORK AND NURSING**: A practical Manual of Clinical Instruction for Nurses and Students. This book will be found especially useful to beginners, to whom its simple description of elementary principles and treatment in surgery will render it invaluable. By Alexander Miles, M.D. (Edin.), C.M., F.R.C.S.E. Over one hundred illustrations.

LIST OF CHAPTERS.

Section I.—Antiseptic Surgery. Chap. 1. General Principles of Antiseptic Surgery—2. Antiseptic Lotions—3. Antiseptic Powders and Unguents—4. Materials for Dressings—5. Lotion Basins—6. Ward Table and Dressing Tray—7. Antiseptic Dressing—8. A Spray Dressing—9. Management of Surgical Operations—10. The Lotion Table—11. Dressings Table—Anæsthetics—12. Preparation of Patient for Operation —13. Treatment after Operation—14. Nursing of Special Cases after Operation.

Section II.—The Use of Rest in Surgery. 15. Extension Apparatus—16. Plaster and Starch Cases—17. Appliances for Joint Affections —18. Appliances for Fractured Bones.

Section III.—Bandaging. 19. Bandaging—20. Special Bandages—21. Special Bandages (*continued*)—22. Special Bandages (*continued*).

Section IV.—Surgical Instruments and Appliances. 23. General Instruments—24. Instruments for Hæmorrhage—25. Knives—26. Saws —27. Bone Instruments—28. Instruments used in Trephining—29. Mouth Instruments, etc.—30. Intestinal Instruments—31. Urethral Instruments—32. Lithotomy and Lithotrity Instruments—33. Laryngeal Instruments—34. Aural, Nasal, and Ophthalmic Instruments—35. Gynæcological Instruments.

"Will furnish much-needed guidance to the student and the nurse in the early stages of their apprenticeship."—*Times.*

"This is a fully illustrated handbook for the use of junior students of medicine and nurses, which should prove of very great value, Dr. Miles being unquestionably an authority on the subject of which he writes."—*Publishers' Circular.*

---

FOR USE IN HOSPITAL AS WELL AS PRIVATE NURSING.

Demy 16mo. (for the apron pocket), 72 pp., strong boards, Second Edition, price 6d., or 4s. 6d. per dozen (post free).

**"THE HOSPITAL" NURSE'S CASE BOOK**: A Book of Tables specially prepared for use by Nurses in the Ward and in the Sick-Room. Containing Twenty-six complete Tables, each being ruled to last one week, for keeping an exact record of a Patient's condition, including notes on Temperature, Pulse, Respiration, Motions, Age, Sex, Name, Address, Occupation, Treatment, History, Name of Medical Attendant, Number of Case, and Date of Admission.

Nurses should combine together and order parcels of over a dozen to profit by the special discount terms—*i.e.*, 4s. 6d. per dozen (post free).

---

Third Edition, enlarged and partly re-written, cloth gilt, crown 8vo., about 200 pp., price 2s. 6d.

**HOSPITAL SISTERS AND THEIR DUTIES.** By Eva C. E. Lückes, Matron to the London Hospital. Every matron, sister, or nurse who aspires to become a sister should read the valuable advice and information contained in this book. The author's experience as matron of London's largest hospital peculiarly qualifies her to speak on this subject.

"We cordially recommend the work to all hospital authorities as well as to the class for whose special benefit it is more immediately intended."—*Daily Chronicle.*

"On every page we find evidence of Miss Lückes' thorough acquaintance with all the details of the nursing department of hospital management." —*Lancet.*

"It is well worthy of study for those to whom it is addressed, and for all who are interested in the matter of nursing."—*New York Medical Journal.*

Demy 8vo., cloth gilt, pp. 260, price 3s. 6d.

**ART OF FEEDING THE INVALID.** By a Medical Practitioner and a Lady Professor of Cookery. Full descriptions of the various ailments, together with the principles upon which they should be dietetically treated, followed by hundreds of original recipes for sick-room cookery.

"Its design is excellent."—*Lancet.*
"A useful book."—*British Medical Journal.*

---

Demy 8vo., 200 pp., Fourth Thousand, profusely illustrated with Copyright Portraits and Drawings, price 2s. 6d., post free.

**HOW TO BECOME A NURSE: And How to Succeed.** Compiled by Honnor Morten, Author of "Sketches of Hospital Life," "The Nurses' Dictionary," &c. A complete collection of information of every kind such as persons desiring to enter nursing can want to know. General advice, useful hints, and full lists of hospitals, nursing schools, and other institutions to which intending nurses can apply for admission as probationers. Also terms, fees, and every other detail.

"A compendium of authentic practical information, which cannot fail to be of service."—*Times.*

"An admirable little manual for those who think of entering upon the profession of nurse, and also for those who, having entered upon it, wish to succeed. It is well arranged, and the information given is detailed and thorough."—*Daily Graphic.*

"The author speaks with the authority of experience and with the advantage of being a shrewd observer, who is, moreover, skilled to convey her ideas in clear and forcible language."—*Daily News.*

"There is no guide we know of which contains in so small a compass more useful or practical information."—*British Medical Journal.*

"Thoroughly accurate and reliable."—*Young Woman.*

"Those wishing to become nurses will find much excellent advice and information of every description pleasantly given. The numerous illustrations make the book very attractive, it is well printed, and the contents so admirably arranged that inquirers can easily find the information they are in want of."—*Nursing Notes.*

---

PUBLISHED THIS DAY.

Second Edition, enlarged, demy 8vo., profusely illustrated, nearly 200 pp. cloth. Important Text-Book for Asylum Attendants, price 2s. 6d. (post free).

**MENTAL NURSING**: A Text-book specially designed for the instruction of Attendants on the Insane. By William Harding, M.D. The want of a complete book for the instruction of Asylum Attendants has been long felt, and it is with confidence that the publshers recommend this work to the managers of all Institutions for the Insane.

The Contents are as follows:—Chap. 1. Elementary Anatomy and Physiology—2. Practical Lessons from preceding Chapter for Asylum Nurses—3. Nervous System—4. Insanity and its forms from the Nurse's point of view—5. Ventilation of Wards—6. Warmth—7. Cleanliness, personal and ward—8. Food and Feeding of Patients, etc.—9. Management of Patients (*a*) on admission, (*b*) general, (*c*) suicidal, (*d*) epileptic, (*e*) general paralytic, (*f*) idiot—10. Epileptics, General Paralytics, and Idiots—11. Things to be Observed and Reported—12. Entertainments—13. The Charge Nurse—14. The Night Nurse—15. The Sick Room, General Remarks, Bed Sores, Wet Pack, Dry Pack, Hot Bath, &c.—Catheter, &c.—16. Hospital and Asylum Nurses—17. Puerperal Insanity —18. Hysteria—19. Nursing the Insane in Private Houses.

OPINIONS OF THE PRESS.

"We have seldom seen instructions to nurses put in so clear and concise a manner, and we think the book should be in the hands of every attendant on the insane. It is, indeed, so good that most junior medical officers in asylums will find it full of the most useful hints."—*Medical Chronicle.*

"We have little hesitation in recommending it as the most suitable and most thorough course of Lectures on Asylum Nursing that has hitherto been published. We do not propose to review the book at any length, but we have especially to commend the first two chapters."—*The Caledonian Medical Journal*, April, 1893.

"Nothing could be better devised to serve as a text-book for those whose duty it is to attend to lunatics."—*Local Government Journal.*

---

London: THE SCIENTIFIC PRESS (Limited), 428, Strand, W.C.

### ACCOUNT BOOKS FOR INSTITUTIONS.

Designed by HENRY C. BURDETT.

Ruled in accordance with the uniform system of accounts advocated by the Metropolitan Hospital Sunday Fund, and prepared for the convenience and assistance of Secretaries who present annual returns for participation in the Hospital Sunday Fund Grants.

These Books are ruled so that the various descriptions of Receipts and Expenditure, &c., may be entered uniformly under the special headings and in the columns prepared for them.

There are TWO SERIES, No. 1 being for the larger Institutions, and No. 2 for Cottage Hospitals and Small Institutions. The sets are sold complete, at a specially reduced price; or each book may be had separately, at the prices set out below:—

### SERIES No. 1.—FOR HOSPITALS AND INSTITUTIONS.

(Any of these Books may be purchased separately; a reduction is made if the complete set is taken.)

1. Analysis Journal. Royal, 350 pp. Divided into seven sections, with parchment tags. Headings as follows:—
   A. Maintenance.
   1. Provisions. 5. Rent.
   2. Surgery & Dispensary. 6. Salaries.
   3. Domestic. 7. Miscellaneous Expenses
   4. Establishment Charges.
   B. Administratation.
   1. Management. 2. Finance.
   (Uniform with Headings in Income and Expenditure Table.) Each Heading is ruled with pages containing 15 columns. Price £3.
2. Cash Book. Foolscap, 300 pp., printed Headings, specially ruled. Price £1.
3. Cash Analysis and Receipt Book. Foolscap, extra width, 300 pp., ruled, with 15 columns, printed Headings identical with Income and Expenditure Table. Price £1 5s.
4. Secretary's Petty Cash Book. 300 pp., foolscap, ruled with 13 columns, printed Headings identical with Income and Expenditure Table. Price £1.
5. List or Register of Annual Subscribers. Double foolscap, 300 pp., ruled with 10 columns, printed Headings for the years 1893-1900 inclusive. Price £1 15s.
6. Alphabetical Register Book. Foolscap, 300 pp., ruled, with date, name and address, and cash colums, cut into alphabetical sections, with totals at end for annual balances. Price £1 6s.
7. Subscription Register. Foolscap, ruled with 8 columns, printed Headings for date when subscriptions become due, name and address, and cash columns for years 1893 to 1898 inclusive. Price £1 1s.
8. Linen Register. Foolscap long quarto, 300 pp., ruled with columns for Stock, Condemned, Remaining, and Issued Linen, also total in Ward and Remarks, also printed lists of Hospital Linen. Price 8s. 6d.
9. Income and Expenditure Table. Basis of uniform system. For yearly balances and statements. Royal. Price 6d. per sheet, or 5s. per dozen.
10. Special Appeal Account Table. Foolscap. Price 3d. per sheet, or 2s. 6d. per dozen.

Price of Set Complete, £10 Net.

### SERIES No. 2.—FOR COTTAGE HOSPITALS AND SMALLER INSTITUTIONS.

1. Cash Analysis, Receipt, and Expenditure Book. Double foolscap, 300 pp., ruled with 18 columns, printed Headings identical with Income and Expenditure Table, specially prepared for Cottage Hospitals and Small Institutions. Price £1 15s.
2. Secretary's Petty Cash Book.—300 pp., foolscap, ruled with 13 columns, printed headings identical with Income and Expenditure Table. Price £1.
3. Income and Expenditure Table. Basis of uniform system. For yearly balances and statements. Royal. Price 6d. per sheet, or 5s. per dozen.
4. Linen Register. Foolscap, 300 pp., ruled with columns for Stock, Condemned, Remaining, and Issued Linen, also total in Ward and Remarks, also printed lists of Hospital Linen. Price 8s. 6d.
5. List or Register of Annual Subscribers. Foolscap, 300 pp., ruled with columns for the years 1893-1900 inclusive. Price £1.
6. Special Appeal Account Tables. Foolscap. Price 3d. per sheet, or 2s. 6d. per dozen.

Price of Set Complete, £4 Net.

The books are all uniformly bound in half basil, with gilt letterings, and are ruled on best paper, and in every way prepared for practical use at the offices of Institutions.

### ACCOUNTS, AUDIT, AND TENDERS; the Uniform System of,

**For Hospitals and Institutions, with Certain Checks upon Expenditure.** Also the Index of Classification, Compiled by a Committee of Hospital Secretaries, and adopted by a General Meeting of the same, 18th January, 1892. And certain Tender and other Forms for securing Economy. By HENRY C. BURDETT. Demy 8vo, cloth, 6s.

This is a hand-book to the account books, explaining their nature and uses, the method of keeping them, and the general principles upon which they were designed.

All having to do with hospital accounts will find much valuable information in this work.

Secretaries making up returns for participation in the Donations of the Metropolitan Hospital Sunday Fund will find it invaluable. It will be of special interest, also, to the officials of all Institutions—Treasurers, Secretaries, and Auditors—as it expounds practically the Uniform System, which is now being widely adopted as a means of saving labour and of bringing all systems of accounts into uniformity.

"This useful and practical little book. No one interested in the hospital question can treat with indifference anything which falls from Mr. Burdett. . . . A uniform system of keeping their accounts. . . . Would be a boon to hospitals generally."—*British Medical Journal.*

London: The Scientific Press (Limited), 428, Strand, W.C.

